# Preoperative cerebrospinal fluid biomarkers may be associated with postoperative delirium in patients undergoing knee/hip arthroplasty: the PNDABLE study

**DOI:** 10.1186/s12877-023-03943-w

**Published:** 2023-05-10

**Authors:** Yanan Lin, Nannan Yu, Xu Lin, Xiyuan Deng, Fanghao Liu, He Tao, Rui Dong, Bin Wang, Yanlin Bi

**Affiliations:** 1 Department of Anesthesiology, Qingdao Hospital, University of Health and Rehabilitation Sciences (Qingdao Municipal Hospital), Qingdao, Shandong province China; 2 Cadre Health Department, Qingdao Hospital, University of Health and Rehabilitation Sciences (Qingdao Municipal Hospital), Qingdao, Shandong province China; 3 Department of Anesthesiology, Dalian Municipal Central Hospital, Dalian University of Technology, Dalian, Liaoning province China; 4 Department of Anesthesiology, Drum Tower Hospital, Nanjing University Medical School, Nanjing, China

**Keywords:** Delirium, Aged, Biomarkers, Predictive factors, cerebrospinal fluid

## Abstract

**Background:**

In the global aging population, the incidence of postoperative delirium (POD) is increasing. Therefore, finding its effective predictive tools becomes crucial. We aimed to identify potential Cerebrospinal fluid (CSF)biomarkers for POD.

**Methods:**

A total of 825 patients undergoing knee/hip arthroplasty under combined spinal-epidural anesthesia were selected. The patients were aged 40 to 90 years with American Society of Anesthesiologists physical status I~II. The Mini-Mental State Examination was completed 1 day before the operation. CSF was extracted after successful spinal-epidural combined puncture, and α-synuclein (α-syn), amyloid beta_40_ (Aβ_40_), amyloid beta_42_ (Aβ_42_), t-Tau, phosphorylated Tau (p-Tau), progranulin (PGRN) and soluble triggering receptor expressed on myeloid cells 2 (sTREM2) in the CSF were measured by enzyme-linked immunosorbent assays (ELISA). The patient’s operation time, anesthesia time, intraoperative blood loss and fluid input were also recorded. After the operation, the occurrence rate and severity of POD were determined by the Confusion Assessment Method and the Memorial Delirium Assessment Scale (MDAS), respectively. Patients were categorized into POD group and non-POD group. Logistic regression analysis was performed on the indicators with statistically significant differences, and the area under the ROC curve (AUC) was used to estimate the predictive accuracy of the biomarkers for POD.

**Results:**

A total of 92 patients developed POD and the incidence of POD was 11.15%. The results of the multivariable logistic regression showed that CSF t-Tau (*P* = 0.004, OR = 1.006, 95%CI 1.002~1.009) and α-syn (*P* = 0.004, OR = 1.001, 95%CI 1.000~1.001) were positively associated with the occurrence rate of POD, while Aβ_42_ (*P* < 0.001, OR = 0.989, 95%CI 0.986~0.993), CSF PGRN (*P* = 0.002, OR = 0.999, 95%CI 0.999~1.000), Aβ_42_/ t-Tau (*P* < 0.001, OR = 0.181, 95%CI 0.102~0.319) and Aβ_42_/p-Tau (*P* < 0.001, OR = 0.617, 95%CI 0.526~0.725) were inversely proportional to the occurrence of POD. ROC curve analysis indicated that Aβ_42_/t-Tau (AUC = 0.823), CSF Aβ_42_ (AUC = 0.813), Aβ_42_/p-Tau (AUC = 0.810), α-syn (AUC = 0.644) and PGRN (AUC = 0.638) could predict the occurrence rate of POD. The combination of all these biomarkers showed a greater AUC(0.896) than using any of them alone.

**Conclusions:**

CSF Aβ_42_, PGRN, α-syn, Aβ_42_/t-Tau and Aβ_42_/p-Tau might be associated with the occurrence rate of POD in patients undergoing knee/hip arthroplasty.

**Trial registration:**

Clinical Registration No. ChiCTR2000033439.

## Introduction

In the Diagnostic and Statistical Manual of Mental Disorders (DSM), Fifth Edition, postoperative delirium (POD) is a disorder in attention, awareness, and cognition that fluctuates wildly over a short period after the operation [1]. It has been reported that the incidence of POD after different surgical procedures ranges from 10%–60% [2], and the POD incidence after knee and hip arthroplasty is 16.5% [3], POD may lead to adverse outcomes such as increased incidences of postoperative complications, longer hospital stays, increased medical costs, and an increased postoperative mortality [4, 5]. Therefore, it is of vital importance to explore the effective predictive tools for the occurrence rate of POD.

At present, POD biomarkers mainly consist of brain-derived biomarkers, inflammation-related biomarkers and neurotransmitter biomarkers [6]. As the body fluid that can most directly reflect the changes of nervous system diseases, CSF is vital to the diagnosis and prediction of POD. More and more attention has been paid to POD biomarkers in CSF.

It is well-known that Aβ aggregation and plaque formation are the main pathological mechanisms of Alzheimer’s disease (AD), and these plaques may exhibit deleterious properties. There is considerable evidence that soluble oligomer Aβ is the main deleterious form [7] and some researchers have found that the action on phosphorylation of Tau protein (p-Tau) plays an important role in the occurrence and development of POD [8].

Triggering receptor expressed on myeloid cells 2 (TREM2), a member of the immunoglobulin family, has anti-inflammatory phagocytic properties in myeloid cells [9]. Expression of soluble TREM2(sTREM2)-related genes can reduce the risk of neurodegenerative diseases [10], and it can repair functional deficits in spatial memory and synaptic plasticity by reducing amyloid plaque deposition. Some researchers found that increased CSF sTREM2 in patients with delirium caused microglial cell activation [11], but the relationship between CSF sTREM2 and POD has been scarcely studied.

Progranulin (PGRN) is mainly expressed by neurons and microglia in the central nervous system, and it can be involved in the regulation of cell proliferation and migration, wound repair, inflammation and cancer [12]. PGRN enhances microglia-mediated phagocytosis and protects against Aβ-mediated neurotoxicity and cognitive dysfunction. Apart from POD, PGRN is also a predictor of other neurodegenerative diseases [13].

α-synuclein (α-syn) is the main component of Lewy bodies [14]. As a common type of α-syn, α-syn oligomers are highly neurotoxic. They can be released from nerve cells to impair axoplasmal transport of nerve cells, leading to apoptosis in dopaminergic neurons [15]. Importantly, the accumulation of α-syn oligomers has also been shown to significantly interfere with cognitive function in mice [16], possibly through the deposition of α-syn in the frontal lobes and cerebral cortex. In addition, α-syn can also lead to microglial activation, production of inflammatory cytokines, and ultimately neurodegeneration.

This study investigated the predictive role of preoperative CSF biomarkers in delirium after knee/hip arthroplasty on the basis of the Perioperative Neurocognitive Disorder and Biomarker Lifestyle Study (PNDABLE).

## Materials and methods

### Study design

The PNDABLE study is an ongoing, large-sample cohort study that began in 2018 to explore the pathogenesis, risk factors, and biomarkers of perioperative neurocognitive dysfunction in the Han Chinese population in northern China for early detection, diagnosis, and intervention of PND. This study has important scientific and practical values for establishing a standardized model of PND warning and prevention in China. Cerebrospinal fluid were collected from all participants after written informed consent was obtained from the patients or their legal representatives. This study was registered in the Chinese Clinical Research Registry (Clinical Registration No. ChiCTR2000033439) and approved by the Ethics Committee of Qingdao Municipal Hospital (Ethical Committee N◦ 2020 PRO FORMA Y number 005).

### Participants

Between June 2020 and March 2021 at the Qingdao Municipal Hospital, 900 participants who were scheduled to receive knee/hip replacement surgery under spinal-epidural anesthesia were recruited. The patients, 40~90 years of age, had intact preoperative cognitive function and had sufficient education to complete the preoperative neuropsychological tests were enrolled.

The exclusion standards included: (1) Mini-Mental State Examination (MMSE) scores ≤ 23; (2) ASA III or higher level; (3) Serious psychological disorders and severe systemic diseases; (4) Familial inherited disease;(5) Coagulation dysfunction; (6) Central nervous system infection, head trauma, epilepsy, neurological disorders and so on; (7) Preoperative anxiety, cognitive impairment and postoperative loss to follow-up.

### Cognitive assessments

The day before surgery, patients’ basic cognitive abilities were assessed using the Mini-Mental State Examination (MMSE). An anesthesiologist assessed the postoperative cognitive level using the Confusion Assessment Method (CAM) and Memorial Delirium Assessment Scale (MDAS)[17] at 9:00–10:00 am and 2:00–3:00 pm twice daily on 1–7 days (or before discharge). Several studies have proved that MMSE and MDAS have good reliability and validity in assessing cognitive state [18, 19]. The patients were separated into two groups based on the assessment results: POD group and non-POD group. Additionally, the MDAS was used to quantify the POD severity [20], postoperative pain using the Numerical Rating Scale (NRS).

### Anesthesia and surgery

Patients were interviewed one day before surgery to collect baseline information, including age, gender, ASA physical status, education background, etc. Additional information on comorbidities and medical history was also collected from the patient’s medical records. All procedures were performed by the same surgical team, and we included patients undergoing joint replacement with bone cement. Specific anesthesia procedures and surgical methods, as previously described [21].

### Sample collection

Following the success spinal-epidural subarachnoid puncture, 2 ml of CSF fluid have been taken and transmitted to the lab in 10 mL polypropylene tubes within two hours. All CSF samples were promptly processed at 2000 g for 10 min at room temperature, and they were then kept at 80 °C for additional examination. Enzyme-linked immunosorbent assays (ELISAs) were utilized to measure the concentrations of α-syn, Aβ_40_, Aβ_42_, total Tau (t-Tau), phosphorylated Tau(p-Tau), PGRN and sTREM2 in CSF. All CSF samples were placed on the same batch of plates at random. To eliminate batch variation, all of the antibodies and plates came from the same lot. Additionally, the CVs within and between batches were each less than 5% and 15%, respectively. All experimental procedures were carried out by researchers who were unaware of the identities of the patients.

### Statistical analysis

SPSS statistical software, version 25.0 (SPSS, Inc., Chicago, IL, USA), and Medcalc software (version 20.0.1, Ostend, Belgium) were used for data analysis. The Kolmogorov -- Smirnov method is used to verify whether the measurement data is normally distributed, and these data were expressed as mean ± standard deviation (SD) for normally distributed continuous variables, or the median and interquartile range (M, P25-P75) for nonnormal distributions. The independent two-sample T test was used to compare the normal distribution data between groups, while non-normally distributed data were compared using Mann-Whitney U test. Counting data were expressed in frequency and percentage, and intergroup comparison was conducted using Chi-square test or Rank Sum test. Significant variables were included in univariate regression analysis. And then multivariable logistic regression analysis analyzed the relationship between the POD-related indicators and POD. The area under the Receiver Operating Characteristic Curve (ROC) was used to predict the accuracy of the associated factors in the diagnosis of POD. The sensitivity and specificity were also calculated. Binary logistic was taken to perform step by step Logistic analysis and produce the predictor variables of combination, PRE. And the new variation PRE was considered as examined variable, dependent variable was POD, calculate the combination AUC.

Considering that the outcome variable should be greater than 10 times of the logistic regression analysis variable [22], and that the previous study of our research group showed a POD incidence of about 12% [23], and a loss to follow-up rate of 20%, at least 900 patients should be included (Included patients = 9*10/0.12*1.2).

## Results

### Participant characteristics

**Fig. 1:**
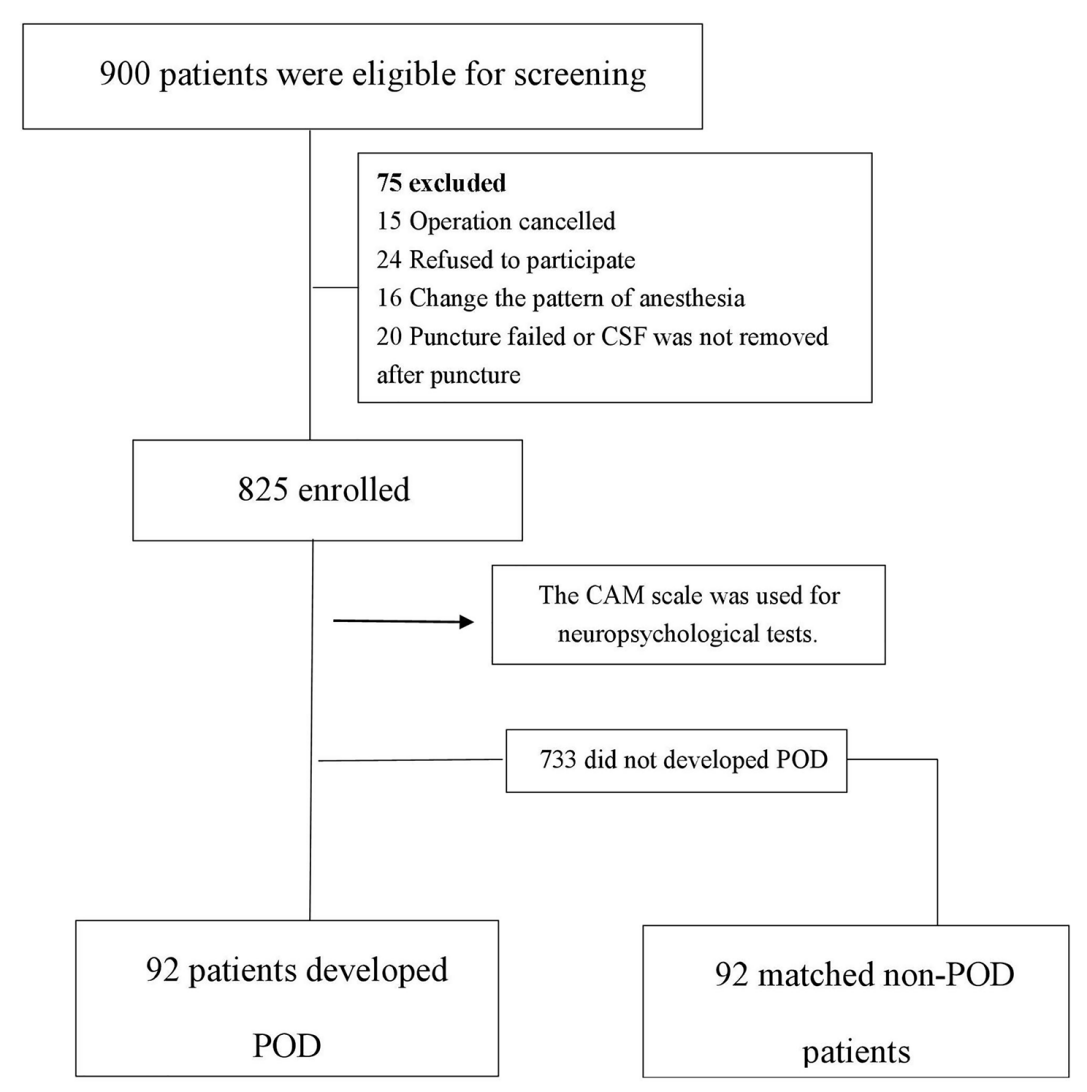
Flow diagram

Among the 900 eligible patients, a total of 825 patients were finally included in this study. In the 825 patients, there were 92 POD cases, with an incidence of 11.15%, as shown in Fig. 1. POD group and the non-POD group were frequency-matched (1:1) on 5 variables, including age, gender, preoperative MMSE score, duration of surgery, and intraoperative blood loss. A non-POD control was randomly selected for each POD case from the non-POD group.

The general conditions of the POD group and the non-POD group were compared. There was no statistical significance in history of diabetes, smoking history, history of hypertension, age, gender, years of education, preoperative MMSE score, duration of surgery, duration of anesthesia, intraoperative blood loss and fluid input (*P* > 0.05). And there was no difference in postoperative Numerical Rating Scale. (Table 1)

**Table 1:** Characteristics of participants

	Non-POD (n = 92)	POD (n = 92)	*P*
Male [n (%)]	54(58.7)	54(58.7)	0.559
History of hypertension, n (%)	36(39.1)	42(45.7)	0.456
History of diabetes, n (%)	18(19.6)	14(15.2)	0.560
Smoking history, n (%)	35(38.0)	32(34.8)	0.759
Age(year)	65(56–71)	66.50(55.50–73)	0.817
Years of education(year)	9(9–12)	9(6–12)	0.353
Preoperative MMSE score	28(27–29)	28(25–29)	0.196
Duration of surgery(min) Duration of anesthesia(min) Intraoperative blood loss(ml) Intraoperative fluid input(ml)	120(110–130) 145(140–160) 120(110–130) 800(800–900)	120(110–130) 140(130–150) 120(110–130) 800(800–900)	0.174 0.413 0.748 0.673
Postoperative NRS score	2.5(2–3)	3(2–3)	0.143

**Abbreviations**: POD, postoperative delirium; MMSE, Mini-mental State Examination; NRS, Numerical Rating Scale

The CSF indexes were compared between the two groups, among which Aβ_42_, t-Tau, PGRN, α-syn, Aβ_42_/ t-Tau and Aβ_42_/p-Tau showed statistically significant differences (*P* < 0.05). And the above indexes were included into the univariable logistic regression analysis. (Table 2)

**Table 2:** Comparison of cerebrospinal fluid related indicators

	Non-POD (n = 92)	POD (n = 92)	*P*
CSF Aβ_40_ (100pg/mL)	56.60(35.82–80.90)	51.77(35.41–73.96)	0.400
CSF Aβ_42_ (pg/mL)	322.4(219.3-454.2)	154.6(114.4-207.8)	< 0.001
CSF t-Tau (pg/mL)	158.6(114.5-203.6)	188.3(127.1-238.7)	0.037
CSF p-Tau (pg/mL)	36.40(27.57–50.29)	39.70(28.79–48.96)	0.690
CSF PGRN (100pg/mL)	27.16(16.70-36.31)	17.54(15.95–26.22)	0.001
CSF sTREM2 (ng/mL)	24.10 ± 12.43	23.11 ± 5.06	0.113
CSF α-syn (100pg/mL)	15.14(8.75–18.43)	17.50(12.91–22.38)	0.001
CSF Aβ_40_/ t-Tau	34.18(24.52–48.12)	25.88(17.17–32.27)	0.951
CSF Aβ_40_/ p-Tau	143.4(99.42–187.2)	103.6(79.0-148.8)	0.175
CSF Aβ_42_/ t-Tau	1.94(1.34–3.16)	0.90(0.65–1.18)	< 0.001
CSF Aβ_42_/ p-Tau	8.43(5.20–12.30)	4.37(3.43–5.53)	< 0.001

**Notes**: Table 2 shows that there were significant differences (*P* < 0.05) between the POD non-POD groups in CSF Aβ_42_, t-Tau, PGRN, α-syn, Aβ_42_/ t-Tau and Aβ_42_/p-Tau, which were included into univariable logistic regression

**Abbreviations**: POD, postoperative delirium; CSF, Cerebrospinal Fluid; Aβ_40_, amyloid beta_40_; Aβ_42_, amyloid beta_42_; t-Tau, total Tau; p-Tau, phosphorylated Tau; PGRN, progranulin; sTREM2, soluble triggering receptor expressed on myeloid cells 2; α-syn, α-synuclein

### Logistic regression analysis of POD risk factors

Univariable and multivariable logistic regression analyses were performed on the indicators with statistical significance in Tables 3 and 4. The results of the multivariable logistic regression showed that CSF t-Tau (*P* = 0.004, OR = 1.006, 95%CI 1.002~1.009) and α-syn (*P* = 0.004, OR = 1.001, 95%CI 1.000~1.001) were risk factors for POD, while Aβ_42_ (*P* < 0.001, OR = 0.989, 95%CI 0.986~0.993), CSF PGRN (*P* = 0.002, OR = 0.999, 95%CI 0.999~1.000), Aβ_42_/ t-Tau (*P* < 0.001, OR = 0.181, 95%CI 0.102~0.319) and Aβ_42_/p-Tau (*P* < 0.001, OR = 0.617, 95%CI 0.526~0.725) were inversely proportional to the occurrence of POD after adjusted for the history of hypertension, the history of diabetes and smoking history.

**Table 3:** Univariate logistic regression analysis

	OR	*P*	95%CI
CSF Aβ_42_(pg/mL)	0.990	< 0.001	0.986~0.993
CSF t-Tau(pg/mL)	1.006	0.004	1.002~1.009
CSF PGRN(100pg/mL)	0.999	0.002	0.999~1.000
CSF α-syn(100pg/mL)	1.001	0.003	1.000~1.001
CSF Aβ_42_/ t-Tau	0.193	< 0.001	0.112~0.335
CSF Aβ_42_/ p-Tau	0.640	< 0.001	0.551~0.744

**Notes**: Table 3 shows that CSF t-Tau and α-syn were risk factors for POD, while CSF Aβ_42_, PGRN, CSF Aβ_42_/ t-Tau and CSF Aβ_42_/ p-Tau were inversely proportional to the occurrence of POD.

**Abbreviations**: CSF, Cerebrospinal Fluid; Aβ_40_, amyloid beta_40_; Aβ_42_, amyloid beta_42_; t-Tau, total Tau; p-Tau, phosphorylated Tau; PGRN, progranulin; α-syn, α-synuclein

**Table 4:** Multivariate logistic regression analysis

	OR	*P*	95%CI
CSF Aβ_42_(pg/mL)	0.989	< 0.001	0.986~0.993
CSF t-Tau(pg/mL)	1.006	0.004	1.002~1.009
CSF PGRN(100pg/mL)	0.999	0.002	0.999~1.000
CSF α-syn(100pg/mL)	1.001	0.004	1.000~1.001
CSF Aβ_42_/ t-Tau	0.181	< 0.001	0.102~0.319
CSF Aβ_42_/ p-Tau	0.617	< 0.001	0.526~0.725

**Notes**: The adjustment factors include the history of hypertension, the history of diabetes and smoking history

**Abbreviations**: CSF, Cerebrospinal Fluid; Aβ_40_, amyloid beta_40_; Aβ_42_, amyloid beta_42_; t-Tau, total Tau; p-Tau, phosphorylated Tau; PGRN, progranulin; α-syn, α-synuclein

### Receiver operating characteristic (ROC) curve of POD-related factors

**Fig. 2:**
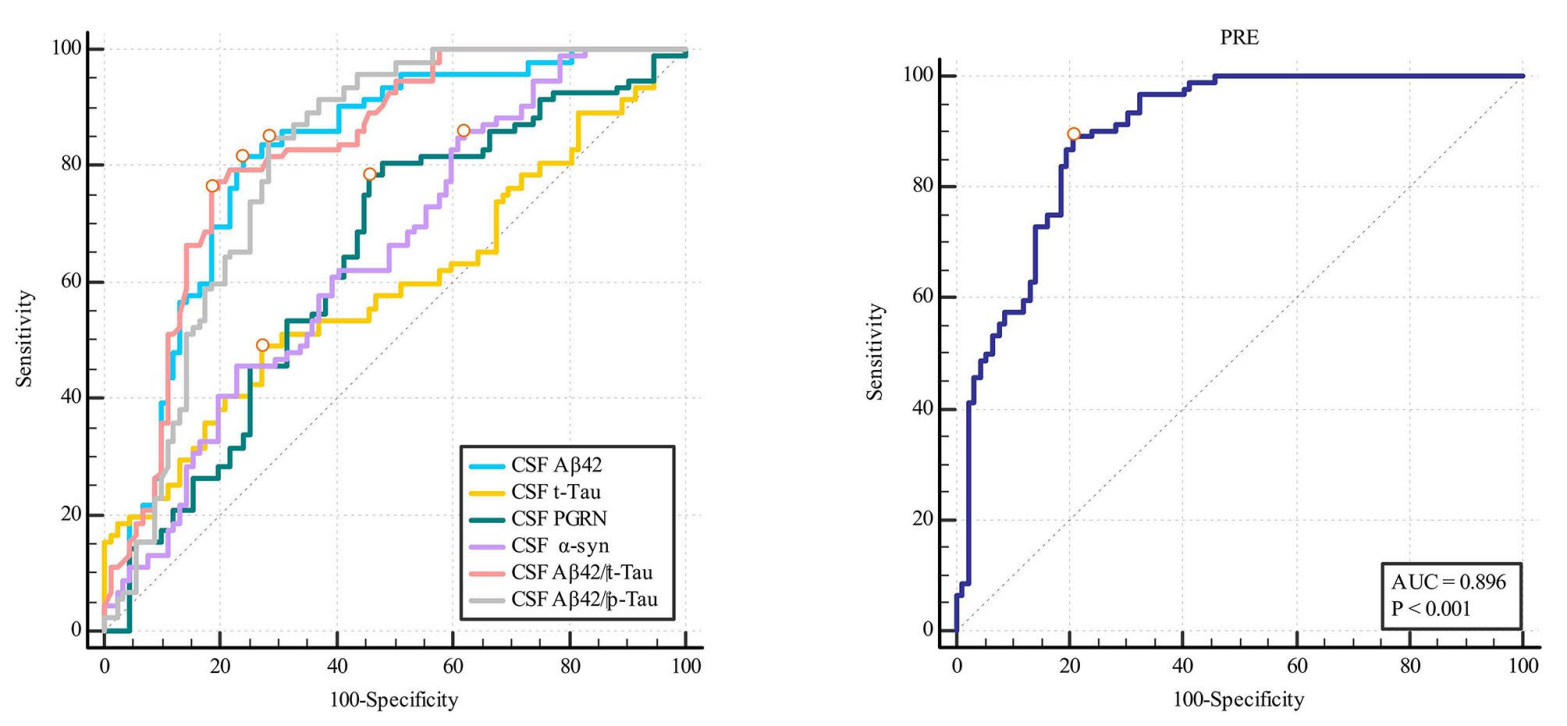
Receiver operating characteristic analysis of associated factors to predict postoperative delirium

ROC curve analysis indicated that Aβ_42_/t-Tau (AUC = 0.823), CSF Aβ_42_ (AUC = 0.813), Aβ_42_/p-Tau (AUC = 0.810), α-syn (AUC = 0.644) and PGRN (AUC = 0.638) could predict the occurrence of POD. If the combination of these biomarkers predicted the occurrence of POD, the AUC(0.896)was larger than that of either biomarker alone. While, t-Tau (AUC = 0.589) have no discriminative power in predicting the occurrence of POD. (Fig. 2)

## Discussion

Of the 825 patients enrolled in this study, 92 developed POD after knee or hip arthroplasty with an incidence of 11.15%. POD is a common neurological complication after surgery, with an incidence ranging from 10% to 60% depending on the surgical procedure [2]. The incidence of POD in this study was lower than previously reported, which may be related to the broad age range of patients included in this study. The research findings show that advanced age is an important risk factor for POD, which may be related to excessive systemic inflammation caused by surgical trauma, impaired circulation, central nervous system immune responses [24], as well as aging-related organ dysfunction and various complications [25].

Consistent with previous studies, this study found Aβ_42_ was decreased in patients in the POD group compared with those in the non-POD group. Aβ aggregation and plaque formation are the main pathological mechanisms of Alzheimer’s disease (AD) and other cognitive diseases [7, 26]. Although these plaques may exhibit deleterious properties, there is considerable evidence that soluble oligomer Aβ is the main deleterious form [7]. Aβ plaque can cause oxidative stress, damaged mitochondria, impaired production of neurons, activation of apoptosis-related proteins, and eventually neuronal cell apoptosis. Research has shown that Aβ can further promote the abnormal phosphorylation of Tau proteins [27], and p-Tau/ Aβ_40_ can better predict the occurrence of neurodegenerative diseases [28] compared with p-Tau alone. But our results indicated that the p-Tau/Aβ_40_ ratio showed no statistically significant differences, while Aβ_42_/ t-Tau was good predictor. Lower Aβ_42_/Tau was associated with poor cognitive function in patients with AD [29]. Studies have confirmed that lower Aβ_42_ levels in cerebrospinal fluid are associated with increased Tau protein levels [30], and preoperative CSF Aβ_42_/ Tau has been found to be not only associated with postoperative delirium, but also negatively associated with its severity, that is, it is negatively correlated with MDAS scores [31].

The results of this study showed that the decreased level of progranulin (PGRN) in CSF could predict the occurrence of POD. Exposure of rat motor and cortical neurons to recombinant PGRN in medium increased the size of neurons and the length of axons [32]. On the contrary, PGRN deficiency promoted microglial transformation from a normal to a pathological state which led to lysosomal dysfunction, neurodegeneration and increased occurrence of neurodegenerative diseases in mice [33]. PGRN-deficient mice were characterized by increased microglial activation and astrocyte proliferation, increased expression of pro-inflammatory cytokines, cell aging, and accelerated ubiquitination [34–36]. It has been confirmed that CSF PGRN is a biomarker of AD [37], frontotemporal dementia (FTLD) [38] and other neurodegenerative diseases. And the results of this study have also showed that CSF PGRN level is related to the incidence of POD, and can be used as a biomarker to predict the occurrence of POD.

The ROC curve analysis also showed that α-syn could predict the occurrence of POD in the elderly patients. The preoperative level of α-syn in CSF of POD group was significantly higher than that of non-POD group. α-syn deposition is a major pathological change in many cognition-related diseases [39], but the exact mechanism underlying the association between α-syn and delirium is still unclear. It has been found that α-syn can promote the aggregation of Aβ protein and Tau protein [40], and further promote the occurrence and development of neurodegenerative diseases. Multiple studies have shown that increased levels of α-syn in cerebrospinal fluid are associated with poorer cognitive function [41–43], which is consistent with the result of this study. If the biomarkers described above are used in combination to diagnose POD, the value is higher than either of them alone.

Our results showed that sTREM2 levels in CSF were higher than non-POD group in POD patients, although this difference was no statistical significance. Previous researches [44, 45] have shown sTREM2 levels correlated with AD progression, and increased sTREM2 is associated with increased Tau levels. In a Norway research, delirium was associated with a higher level of CSF sTREM2 only among those without pre-existing dementia, but the researchers found [11] that, among patients with dementia, there was no group difference. We considered that the results of this study might be due to the wide age range of the patients we included. Another reason may be that all the included patients were of ASA I ~ II, and the general condition of the patients was good. and we may consider further research in the future.

In this study, only patients undergoing knee/hip replacement under spinal epidural combined anesthesia were selected for analysis, without considering the influence of different anesthesia methods on POD. And no specific study was conducted on the mechanisms underlying the associations between changes in CSF biomarkers and POD. And we need to conduct more extensive studies on POD-related proteins in CSF. Although our study included in the statistical model many of the previously reported important potential covariates affecting cognition, the possibility of residual confounders cannot be ruled out. Furthermore, our technique for CSF acquisition is invasive. Future studies will adopt less invasive methods to collect and analyze POD-related biomarkers in blood.

## Conclusion

In conclusion, CSF Aβ_42_, PGRN, α-syn, Aβ_42_/t-Tau and Aβ_42_/p-Tau might be associated with the occurrence rate of POD in patients undergoing knee/hip arthroplasty.

## Data Availability

The raw data supporting the conclusions of this article will be made available by the corresponding author (Prof. Yanlin Bi, Email: pndable2021@sina.com), without undue reservation.
